# Radiation survey around a Liac mobile electron linear accelerator for intraoperative radiation therapy

**DOI:** 10.1120/jacmp.v10i2.2950

**Published:** 2009-04-28

**Authors:** Mario Ciocca, Guido Pedroli, Roberto Orecchia, Andrea Guido, Federica Cattani, Raffaella Cambria, Umberto Veronesi

**Affiliations:** ^1^ Unit of Medical Physics European Institute of Oncology Milano Italy; ^2^ Division of Radiation Oncology European Institute of Oncology Milano Italy; ^3^ Faculty of Medicine Università degli Studi di Milano Milano Italy; ^4^ Scientific Direction European Institute of Oncology Milano Italy

**Keywords:** radiation protection, electrons, mobile linear accelerator, IORT

## Abstract

The aim of this study was to perform a detailed analysis of the air kerma values around a Liac mobile linear accelerator working in a conventional operating room (OR) for IORT.

The Liac delivers electron beams at 4, 6, 8 and 10 MeV. A radiation survey to determine photon leakage and scatter consisted of air kerma measurements on a spherical surface of 1.5 m radius, centered on the titanium exit window of the accelerating structure. Measurements were taken using a 30 cm^3^ calibrated cylindrical ion chamber in three orthogonal planes, at the maximum electron energy. For each point, 10 Gy was delivered. At selected points, the quality of x‐ray radiation was determined by using lead sheets, and measurements were performed for all energies to investigate the energy dependence of stray radiation. The photon scatter contribution from the metallic internal patient‐shielding in IORT, used to protect normal tissues underlying the target, was also evaluated. At seven locations outside the OR, the air kerma values derived from in‐room measurements were compared to measurements directly performed using a survey meter. The results, for a delivered dose of 10 Gy, showed that the air kerma values ranged from approximately 6 μGy (upper and rear sides of the Liac) to 320 μGy (lateral to beam stopper) in the two orthogonal vertical planes, while values lower than 18 μGy were found in the horizontal plane. At 10 MeV, transmission behind 1 cm lead shield was found to be 42%. The use of internal shielding appeared to increase the photon scatter only slightly. Air kerma values outside the OR were generally lower than 1 mGy for an annual workload of 200 patients. Thus, the Liac can safely work in a conventional OR, while the need for additional shielding mainly depends on patient workload. Our data can be useful for centers planning to implement an IORT program using a mobile linear accelerator, permitting radiation safety personnel to estimate in advance the shielding required for a particular workload.

PACS number: 87.55.ne, 87.56.bd

## I. INTRODUCTION

For many years, intraoperative radiation therapy (IORT) has been commonly performed using a conventional linear accelerator located in a radiotherapy treatment vault or, in only a few cases, in a dedicated, shielded operating room (OR).^(^ ^1^ ^–^ ^4^ ^)^ The recent development and rapid growth of dedicated treatment units, namely mobile linear accelerators, have changed the practice of IORT in a significant way, by allowing a larger number of patients to undergo a normal surgical procedure and immediate irradiation without the need for transportation outside the operating theatre.

Mobile linear accelerators are quite compact and designed to operate only in the electron mode up to 10–12 MeV, thus offering the great advantage that they can work in almost any conventional OR, with little or no added shielding, depending on patient workload.^(2)^

Concerning radiation protection, the AAPM recently published recommendations for the implementation of an IORT program using mobile linear accelerators in a non‐dedicated environment.^(2)^ In particular, it recommended that a radiation survey around the ORs used for IORT be mandatory, to ensure that the maximum exposure limits in the adjacent areas are not exceeded.

Despite the increasing number of mobile units installed worldwide, especially in Europe,^(3)^ there have been very few publications on radiation protection issues for these units; most of them are mainly focused on neutron production rather than photon leakage.^(^ ^5^ ^–^ ^8^ ^)^

The aim of this study was to perform a detailed analysis of the x‐ray air kerma rates and the shielding assessment of a Liac (Info&Tech, Roma, Italy, http://www.sordina.com) mobile electron linear accelerator installed in a conventional OR. At our Institute, the Liac is mainly used for IORT to the breast at doses ranging from 12–16 Gy (in case of anticipated boost to the tumour bed and nipple‐sparing mastectomy) up to 21 Gy (exclusive irradiation modality in partial breast irradiation), in all cases prescribed at 90% isodose.^(^ ^9^ ^–^ ^13^ ^)^

## II. MATERIALS AND METHODS

The Liac is a mobile linear accelerator dedicated to IORT in the OR. As already described elsewhere,^(^ ^10^ ^–^ ^12^ ^)^ it delivers electron beams at nominal energies of 4, 6, 8 and 10 MeV and a high dose rate of 4‐31 Gy/minute (depending on beam energy and applicator size). Hard‐docking beam collimation is achieved by means of different PMMA applicators with diameters ranging from 3 to 12 cm, fat‐ended or bevelled. Unlike conventional linear accelerators, no adjustable collimators are used. The nominal treatment source‐to‐skin distance (SSD) is 60 cm. The Liac has no beam bending magnet and employs a thin (80 μm) brass scattering foil to enlarge the original pencil beam. A movable beam stopper, consisting of a 13 cm thick lead shield, 40 cm×40 cm in area, is manually positioned by the operator below the surgical couch to intercept the beam axis. To ensure that the beam stopper is correctly positioned with respect to the beam axis (also in the case where the beam axis is inclined), the Liac is provided with an interlock based on an electronic device, which is able to recognize the position of the center of the beam stopper and check it in the actual setting of the radiation head of the Liac itself.

**Figure 1:**
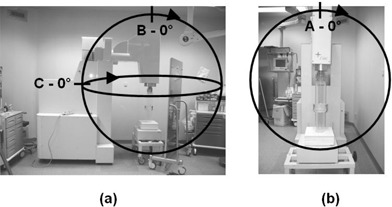
Side (a) and front views (b) of the Liac in parking position inside the operating room. Measurement planes (A, B, C), together with position n. 1 (0°) and direction from position n. 1 forward, are reported (not to scale). In the experimental set‐up, the Liac was positioned to simulate a standard treatment geometry, with the radiation head raised up with respect to the parking position shown. The beam stopper and an example of a movable vertical shield in front of the Liac, as well as an applicator docked to the head, are also shown.

A radiation survey was performed to determine photon leakage and scatter. It consisted of air kerma measurements on a spherical surface of 1.5 m radius centered on the titanium exit window of the accelerating structure, which is close to the scattering foil, with both structures representing the primary sources of photon fluence.^(5)^ Measurements were taken in three orthogonal planes (Fig. 1) every 22.5°, with the exception of one point which could not be tested due to the presence of the Liac stand. The maximum electron energy (10 MeV) and applicator size normally used in clinical practice (10 cm diameter) were used. For each point, monitor units corresponding to 10 Gy dose at the depth of maximum dose and standard treatment distance were delivered. The Liac was positioned in the center of the OR, with the beam axis oriented vertically. Patient scatter in treatment configuration was simulated by using a water‐equivalent slab phantom (30 cm×30 cm wide, 20 cm thick) in contact with the end of the applicator, evaluating the effect of recessing the applicator inside the phantom as negligible in terms of stray radiation. A cylindrical ionization chamber (type 23361, 30 cm^3^ sensitive volume, PTW, Germany), designed to give a fat response over a wide energy range, was utilized, with a 5 mm water‐equivalent buildup cap. The ion chamber was calibrated at a Secondary Standard Dosimetry Laboratory in terms of air kerma under 150 kV x‐ray beams, since it is mainly used for radiation protection measurements in diagnostic radiology. The detector was secured in position by means of an adapted tripod and connected to a digital electrometer (Unidos, PTW). Readings were corrected for ambient temperature and pressure. Three PMMA sheets, each two centimeters thick, were placed between the source and the detector to absorb scattered electrons.^(5)^

Also, at a few selected points, the quality of the combined leakage and scatter was determined by using lead sheets surrounding the ion chamber, while additional measurements were performed for all the electron energies to investigate the energy dependence of stray radiation. The contribution of photon scatter due to the use of metallic internal shielding in IORT (to protect normal tissues underlying the target,^(10)^) was also evaluated by placing, inside the phantom, the largest and thickest available coupled discs (8 cm diameter, 4 mm lead plus 4 mm aluminum thickness, with the aluminum side faced towards the beam) centered on the beam axis and at the depth of dose maximum (13 mm).

**Figure 2:**
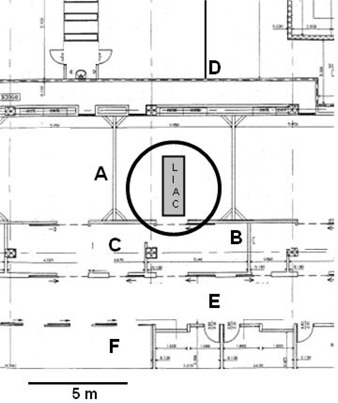
Floor plan of the operating room and the adjacent areas. The typical working position of the Liac (outlined) as well as the points (A to F) where the air kerma values were calculated, are shown.

Finally, expected air kerma values were calculated outside the OR by simply applying the inverse square law to the values measured at 1.5 m in that direction. The calculated values were then compared to those derived by air kerma rate measurements carried out in the areas adjacent to the OR on the same floor (Fig. 2) and one floor below the Liac. In particular, point A was located in the adjacent OR (1 m from the wall), B at the Liac treatment console, C at the centre of the operators' washing room, D inside a storage room (normally accessible to personnel), E along the corridor connecting all the ORs, and F in the patient preparation room. At point G (one floor below), the expected air kerma was calculated by applying an approximate attenuation factor of 10 to take into account the shielding effect of the concrete floor, 22‐cm thick (Note: see Fig. A.1a. in the NCRP report No. 151^(14)^). All measurements were performed at the height of 1.5 m from the floor, using a portable ion chamber survey meter (RPO‐50, 349 cm^3^ sensitive volume, Victoreen, USA) calibrated in terms of air kerma rate under Cs‐137 γ ray beams. As stated by the manufacturer in the user's manual, this type of detector shows a fat energy response from high‐energy down to about 100 keV x‐rays. Air kerma rates were converted into air kerma values corresponding to annual workload of 200 patients irradiated at a dose of 20 Gy each. This was accomplished by taking into account the dose rate produced by the Liac at 10 MeV, approximately equal to 20 Gy per minute (i.e. by multiplying raw rate data by the workload in hours of beam‐on time per year, 200/60=3.33 h/y).

The overall uncertainty of measurements carried out with the 30 cm^3^ ion chamber was around ±7% (1 S.D.), estimated by combining in quadrature the following parameters: air kerma calibration (2.5%), positioning uncertainty (3 cm, 4%), energy dependence (4%) and short‐term reproducibility (0.2%) of the ion chamber, air density correction (0.5%) of the reading, electrometer calibration (0.2%) and the Liac output calibration (2%). For the portable survey meter, the uncertainty was about 13%, taking into account the air kerma rate calibration (6.5%), energy dependence (10%), and dose rate constancy of the Liac (5%).

## III. RESULTS

The results of air kerma measurements performed using 10 MeV at the distance of 1.5 meters around the Liac are reported in Table 1.

**Table 1:** Measured air kerma values, expressed in μGy per 10 Gy delivered dose, at 1.5 m from the exit window of the accelerating structure of the Liac, using the maximum beam energy (10 MeV), in the three planes shown in Fig. 1.

*N*.	*Angle (°)*	*Plane A*	*Plane B*	*Plane C*
1	0	13.5	13.5	7.2
2	22.5	9.0	9.9	16.2
3	45	7.2	6.4	17.6
4	67.5	11.5	10.2	15.0
5	90	16.8	13.7	16.8
6	112.5	22.8	24.7	13.7
7	135	38.3	36.8	12.5
8	157.5	196.4	117.7	11.0
9	180	51.5	51.5	13.7
10	202.5	274.3	321.6	12.1
11	225	69.0	n.m.^a^	11.6
12	247.5	26.7	15.0	13.1
13	270	15.4	7.2	15.4
14	292.5	14.1	5.7	13.0
15	315	7.7	6.2	15.6
16	337.5	8.9	9.8	14.1

a n.m.=not measurable

Additionally, air kerma values measured again at 1.5 meters without the beam stopper and along the radiation beam axis (plane A, angle 0°) were 1028.2, 716.5, 430.9, and 292.8 μGy at 10, 8, 6, and 4 MeV, respectively. Corresponding calculated values below the beam stopper were the following: 51.5 (5% of the value measured without beam stopper), 38.9 (5%), 21.2 (5%) and 12.7 μGy (4%) for 10, 8, 6 and 4 MeV, respectively.

The dependence of stray radiation on beam energy was also investigated at a point outside the primary beam path (plane B, angle 157.5°, also at 1.5 m from the Liac) and air kerma values at 8, 6 and 4 MeV were found equal to 90%, 73% and 69%, respectively, of that measured at 10 MeV. At the same location, in relation to the quality of photon leakage and scatter at 10 MeV, we found that the air kerma behind 1 cm of lead attenuator was 42% of the value measured without shield, indicating that the first half‐value layer is approximately equal to 8 mm of lead. Finally, the utilization of the metallic disk (lead and aluminum) as an internal shielding inside the radiation field showed an increase of measured air kerma of 21% at 10 MeV (142.2 vs 117.7 μGy) – this energy representing the worst case scenario.

Expected and measured air kerma values outside the OR for the previously specified annual workload, are reported and compared in Table 2. At point A, the air kerma was also measured using a thick PMMA phantom in front of the ion chamber to guarantee removal of electron component.

**Table 2:** Air kerma values determined for annual workload of 200 patients treated at a dose of 20 Gy each, in 6 selected points (A to F, see Fig. 2) in the areas adjacent to the operating room where IORT is delivered and on the floor below the Liac (point G). Comparison between expected values from in‐room measurements (column n. 4) and direct measurements using the portable survey meter (column n. 6) is also reported (see text for details).

*Point*	*Raw value from Table* 1 *(μGy) (and its position* ^a^)	*Distance from the source (m)*	*Expected kerma (mGy/y)*	*Measured raw value (μGy/h)*	*Measured kerma (mGy/y)*	*Kerma difference (%)*
A	16.8 (C 5)	4.2	0.86	382 (287^b^)	1.27 (0.95^b^)	+48 (+10 ^b^)
B	15.6 (C 15)	4.5	0.69	203	0.68	−1
C	17.6 (C 3)	4.7	0.72	227	0.76	+10
D	12.1 (C 10)	5.8	0.32	60	0.20	−38
E	14.1 (C 16)	6.5	0.30	84	0.28	−7
F	11.7 (C 0÷1)	8.6	0.14	25	0.08	−43
G	321.6 (B 10)	4	1.81^c^	358	1.19	−34

a plane of measurement and point number (see Table 1)

b solid phantom (6 cm thick) in front of the detector

c attenuation factor of 10 due to concrete floor taken into account (see text)

## IV. DISCUSSION

An analysis of photon leakage and scatter from a mobile electron linear accelerator for IORT using a Mobetron (Intraop Medical, Inc., USA) was published in 2001 by Daves and Mills.^(5)^ However, those data cannot be extended to a Liac, due to the structural differences between the two machines. In particular, the Mobetron uses two colinear accelerators, being the area where they meet a critical point of potential leakage.^(5)^ Moreover, the Mobetron is provided with a lead self‐shielding and soft‐docking beam collimation that is achieved by means of metallic applicators. In addition, all data reported in that work were averaged over all electron energies (4, 6, 9, 12 MeV), assuming an equal workload contribution. However in 2006, the AAPM TG‐72 clearly specified that the radiation survey for IORT mobile machines *must* be performed using the highest electron energy.^(2)^ We proceeded to compare the air kerma measurements on a Mobetron from the Daves and Mills study and a Liac (present study). In order to make the two data sets consistent, measurements originally reported for the Mobetron at 2 meters from the scattering foil have been first recalculated at 1.5 meters using the inverse square law. In general, air kerma values found for the Mobetron are lower than those reported in this investigation. In particular, the average kerma measured in the two vertical planes was 26.4 μGy for the Mobetron and 46.1 μGy (75% more) for the Liac. This difference is only partially explained by the different nominal electron energy (7.8 MeV as an average value versus 10 MeV). The main reason for that difference could be found in the self‐shielding of the Mobetron, but unfortunately no details on this issue are given in the report.^(5)^ As expected, on both machines the maximum and minimum kerma values respectively occur laterally to the beam stopper (187 μGy for the Mobetron versus 322 μGy for the Liac) and in the opposite direction, towards the ceiling (3.6 μGy versus 6 μGy, respectively). In the horizontal plane, the difference in the average air kerma between the two machines was lower (11.7 μGy versus 13.7 μGy).

Regarding the radiation quality, the first half value layer measured on the Mobetron at 9–12 MeV (13–14 mm lead) appears higher than the value measured on the Liac at 10 MeV (8 mm lead), perhaps due to radiation hardening effect by the Mobetron self‐shielding.

In our study, the effect of metallic internal shields on photon scatter has been documented for the first time. In our opinion, their use in IORT produces a net benefit – the 20% increase in measured air kerma being more than counterbalanced by the optimal dose sparing of underlying normal tissues (for example, in breast cancer: ribs, pectoral and cardiac muscles, lung).

Concerning the comparison between expected and measured kerma values outside the OR where the Liac is operated (Table 2), differences within ±10% were found in three cases (points B, C, E). In the adjacent OR (point A), close agreement was found only after removal of electron scatter, indicating that in our clinic the thin wall between ORs is not sufficient to stop it. Therefore, vertical mobile shieldings (1 cm lead) we normally position during IORT treatments around the surgical table aim not only to reduce by roughly half the exposure due to photon leakage and scatter, but also to reduce electron scatter. The findings of the measured electrons in the adjacent OR at a short distance from the Liac can be explained by the following discussion. For 10 MeV, the electron radiation transmitted through the PMMA wall of the applicator (5 mm thickness) is relatively high (from a radiation protection point of view), being about 7% of the dose measured along the beam axis and at the depth of $d_{\max}$; such a very energetic electron component is able to travel in air for several meters and 4–5 cm of water are needed for stopping it almost completely. In our specific case, the materials and thickness of the wall between adjacent ORs seem not adequate enough to do it. On the contrary, this effect was not seen at points B and C although the distances are similar, due to a different type of wall and the existence of a metallic sliding door.

Differences around 40% were found at larger distances from the Liac (points D and F) and can be explained by the attenuation effect of multiple walls and metallic doors, not taken into account in the calculation of expected kerma. Similarly, at the floor below the Liac (point G), measured kerma was considerably lower than the expected one, suggesting an underestimation of the attenuation factor assumed in the calculation for the concrete floor.

In view of the previous evaluations, the two data sets (i.e. the expected and the measured air kerma values) may then be considered consistent. While the expected air kerma data can be used as a preliminary assessment of environmental radiation protection issues (workload restrictions, need for shielding, classification of workers, and adjacent areas), the measured data serve as a mandatory radiation survey to check building materials and validity of exposure estimations under the user's own conditions.

Finally, our data appear to confirm the indication of 3–4 patients per week (i.e. 200 patients per year) treated at the IORT dose of about 20 Gy using a mobile linear accelerator as a safe workload in an existing OR with little or no added shielding, as already reported.^(2)^ ^,^ ^(5)^ In addition to patient treatment, daily warm‐up irradiation and constancy checks have to be carefully planned to ensure that maximum exposure limits are not exceeded. For example, at our Institute the policy has been adopted of performing those irradiations outside normal working hours.

## V. CONCLUSIONS

This study confirmed that the Liac can safely work in a conventional OR, while the need for added shielding mainly depends on patient workload. The air kerma values reported in the present investigation can be quite useful for those centers planning to implement an IORT program using a mobile linear accelerator, to estimate in advance shielding requirements and permitted workload.

## ACKNOWLEDGEMENTS

The authors are grateful to AIRC (Italian Association for Cancer Research) and AICF (American Italian Cancer Foundation) for their support of the IORT project at our Institute.
